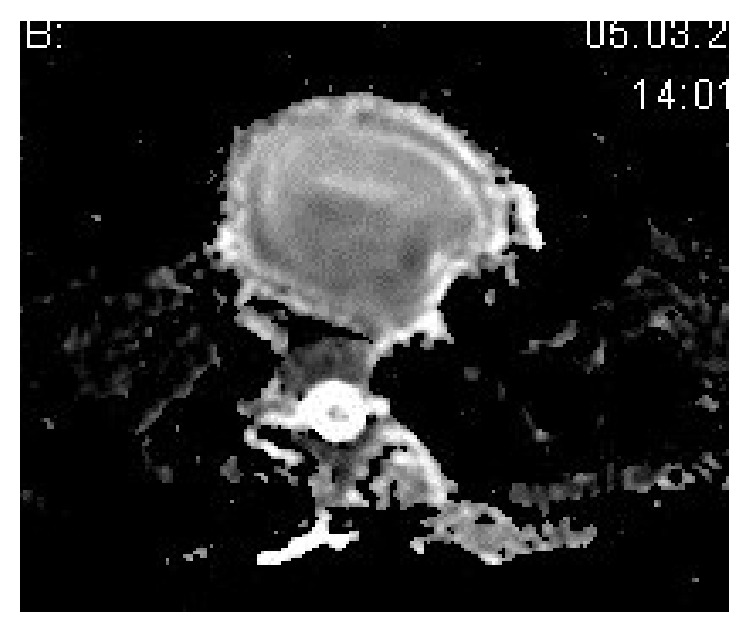# Giant Thyroid Abscess Related to Postpartum Brucella Infection

**DOI:** 10.1155/2015/646209

**Published:** 2015-03-16

**Authors:** Zülküf Akdemir, Erbil Karaman, Hüseyin Akdeniz, Cem Alptekin, Harun Arslan

**Affiliations:** ^1^Department of Radiology, Van Research and Training Hospital, 65000 Van, Turkey; ^2^Department of Obstetrics and Gynecology, Yüzüncü Yıl University, 65000 Van, Turkey; ^3^Department of Radiology, Van Private İstanbul Hospital, 65000 Van, Turkey; ^4^Department of Radiology, Yüzüncü Yıl University, 65000 Van, Turkey

## Abstract

Thyroid gland infection, although rare, may be a life threatening disease. Thyroid abscess, arising from acute suppurative thyroiditis (AST), is a rare clinic condition depending on widespread use of antibiotics. Infection may involve one or both lobes and abscess formation may not be apparent until late stage of the progress of illness. Thyroid left lobe is more often affected than the right one. Brucellosis, especially obvious in endemic areas, is a widely seen zoonosis around the world. Although brucella infection can affect many organs through various complications, thyroid gland infection is rare. We aimed to present ultrasonography (USG) and magnetic resonance images (MRI) of a case with an acute thyroiditis which rapidly developed and grew fast on the left half of the neck during the first postpartum month. As far as we know from literature reviewing, our case is the first case report of a thyroid abscess arising from brucella infection which is developed in first postpartum period with images of ultrasonography and MRI.

## 1. Introduction

Gram-positive organisms such as* Staphylococcus* or* Streptococcus* species are the most widespread pathogenic types but Gram-negative suppurative thyroiditis is rarely reported [1]. Brucellosis is a zoonotic illness creating an important health problem in many regions of the world including Turkey [2].* Brucella melitensis* and* Brucella abortus* are considered the most frequent types for disease in people. Brucella infects people mainly through digestion, scars and cracks on skin, and inhalation of contaminated dusts [3, 4]. Whereas brucellosis is a localized illness causing abortus and sterility in animals, it is a multisystemic illness varying between an illness involving various organs and tissues in humans with clinical symptoms with large spectrum and an acute septicemia [5]. Brucellosis may affect gastrointestinal system, hematologic system, muscle-skeleton system, neurologic system, respiratory system, cardiovascular system, eye, and skin [6]. Acute suppurative thyroiditis causing thyroid abscess is a rare clinical case and it constitutes 0.1–0.7% of all surgically treated thyroid illnesses and less than 1% of all brucella complications [7, 8]. We aimed to present the MRI and USG images of a giant thyroid abscess admitted with acute thyroiditis with rapidly developed and enlarged mass in neck in first postpartum period arising from brucella infection that is rarely seen in the literature.

## 2. Case

**Figure 1:**
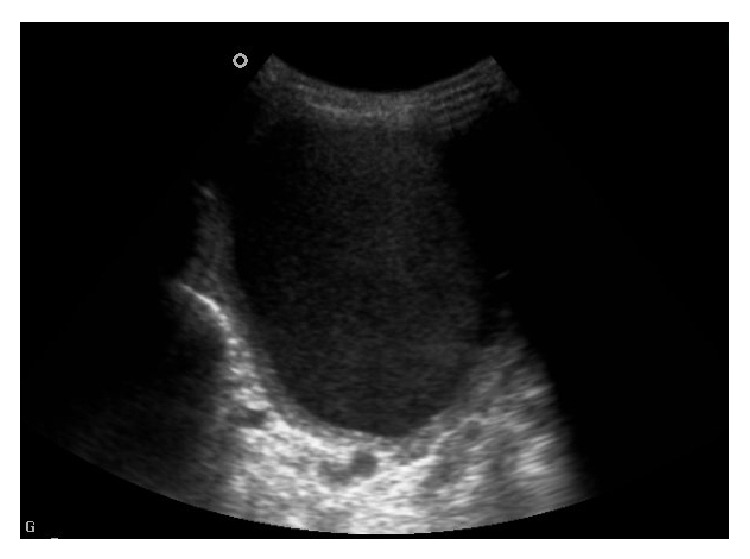
It shows cystic lesion of 10 × 8 cm in lateral left lobe of thyroid gland including internal echoes with some separation and posterior acoustic shadowing in ultrasound examination.

The patient, 25 years of age, referred to our hospital with a fast-growing mass which appeared 3 days ago on the left half of the neck in the first month of postpartum, had complaints of fever, perspiration at night, and tachycardia. Lab analyses showed the following data: low TSH 0,001 (0,35–4,94 *μ*IU/mL), high free T4 2,07 (0,7–1,7 ng/dL), anti-TPO 17,41 (0–5,61 IU/mL), CRP 66,3 (0–5), and sedimentation 56 (1–20). On the USG exam, a cystic mass lesion measuring 10 × 8 cm containing internal echoes, with thick wall, a few thinner septas, and obvious acoustic strength, with unclear relationship with thyroid left gland, and deviated to left with pressure on trachea was seen (Figure 1).

**Figure 2:**
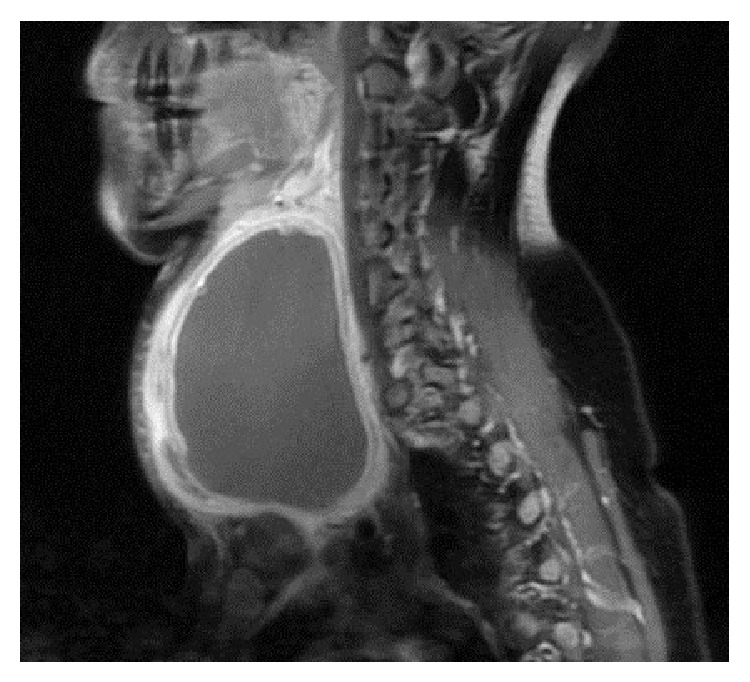


Ultrasound-guided 200 cc hemopurulent liquid aspiration was applied for diagnostic and palliative treatment of patient who had respiratory distress. The pathology of the thin needle aspiration biopsy was considered compatible with the abscess formation containing blood elements, dense neutrophils and leucocytes, many degenerated cells of no clear nature, and dense histiocyte. Examination of the culture of the liquid showed* Brucella melitensis* positive. In serology, tube agglutination test showed 1/1200 titer positive. Later, contrasted neck MRI of the patient was conducted in order to investigate the relationship between the abscess and thyroid gland and neighboring structures. On MRI, a cystic mass lesion, approximately 10.5 × 8.5 × 7 cm, thick-walled, slightly hyperintense on T1-oriented images, and hyperintense on T2-oriented images, with peripheral contrast following intravenous contrasting material injection, containing thin septa, attracted attention (Figure 2).

In addition, on diffusion-oriented series, the diffusion limitation of the cystic content in accordance with abscess existed (Figure 3). The patient had undergone surgery in the surgery department. Thyroid left lobe, isthmus, and pyramidal lobe, together with abscess, were excised on the operation. On postoperative follow-up examination, the patient was free of complaints with the thyroid hormone levels within normal ranges of TSH 2.32 (0,35–4,94 *μ*IU/mL), free T4 1.5 (0,7–1,7 ng/dL), and free T3 3.80 (3,50–6,10 pg/mL) and there was no need for thyroid hormone replacement therapy.

## 3. Discussion

Thyroid infections are rare because of its infection resistant gland capsule, high iodine content, rich blood flow, and extensive lymphatic drainage and it is separated from other structures in neck with fascial planes. When acute suppurative thyroiditis is not treated, it may cause an abscess formation usually triggered by Gram-positive and Gram-negative organisms [1]. Brucellosis is a systemic infectious disease which may affect many organs and systems. The most frequent complication of brucella is bone and joint involvement and it has been reported as 20–85% in different case series [3, 9]. Thyroid gland involvement due to brucellosis was first reported in Kuwait in 1989 [10]. As in our case, thyroid gland involvement is seen as mass on the neck [4, 11]. In addition, as in our case, secondary to the mass, neck pain, difficulty in swallowing, and shortness of breath may be observed. Brucellosis should be excluded in cases of hyperthyroidism and thyroid cancer [11, 12]. The gold standard method in brucellosis diagnosis is culture and it may not be always available due to reasons such as antibiotherapy. In our case, the first liquid aspirated from the abscess was also culture positive. Therefore, the diagnosis is usually conducted with serological methods [13]. In acute suppurative thyroiditis, there is usually sudden-onset pain evolving from anterior neck radiating to chest, mandibular, and ear. Also, unilateral feeling loss, fever, and other symptoms of infection accompany these findings. Gland is painful on palpation and the surface of skin is warm. Although the most frequent reason for thyroid infection is spreading through hematogenous way, the exact way of spreading has not been established [1]. Acute thyroiditis patients are usually euthyroid and thyroid autoantibodies are negative. However, thyrotoxicosis and hypothyroidism have been reported [14]. Our case had thyrotoxicosis.

Thyroidal abscess is more often observed in females [15]. It may be observed as cold or normal nodule on radioactive iodine scan. Radiologic methods should be preferred in order to evaluate abscess diagnosis, its extension to neighboring structures, and its relationship with thyroid gland. As in our case, USG highly possibly supports abscess; MRI, on the other hand, clearly shows the abscess diagnosis and its relationship with neighboring structures. In such cases, reconstructive surgery is needed in order to prevent abscess formation from recurrence.

In conclusion, particularly in brucella-endemic regions such as our country, when mass formation on neck area is encountered, abscess formation secondary to brucella infection should be considered and radiologic methods should be applied for diagnosis and its extension.

## Figures and Tables

**Figure 3:**